# Sanitary Statistics of England

**Published:** 1873-07

**Authors:** T. D. Crothers

**Affiliations:** Albany, N. Y.


*Sanitary Statistics of England.* By T. D. CROTHERS, M.D., of Albany, N. Y.

The Registrar-General's annual summary of births, deaths, and the causes of death, for 1872, in London and other large cities of England, contains many suggestive facts, which are very interesting in view of the recent prominence of sanitary science.

The author discusses the social and economical advantages of large cities, as centres of civilization, showing that there are no evidences of deterioration in the inhabitants; but there is a limit to the growth of every city, similar to the growth of organic forms. This limit of extension depends more upon the species than space or time. Some of these limits may depend upon the supply of water, fuel, and food, or security from enemies, or the attractions or want of attractions for business, health, and pleasure. One prominent cause of the limitation of cities in Europe is their unhealthiness. For two centuries London was literally "a city of plagues," and truly called the "grave of the nation." There is a physical limit to the number of people who can live on a given space. But this varies in London, Edinburg, and on the Continent, where lofty houses, extending up many stories, increase the number of people to a dangerous degree. Happily this system has not prevailed to a great extent. The entire population in England is less than one person to an acre of ground. In twenty of the large towns of the kingdom the population was twenty-nine to the acre. In London it is forty-two per acre. This varies from seven to four hundred and twenty-nine in different districts. The limit of one hundred and fifty seems to have been seldom exceeded during the last ten years, in sections where the population has become denser. In nearly all the sections where the numbers were greater, they have diminished steadily. The well-established law, that the insalubrity of a place increases with the density of its population, and that fevers generated in crowded dwellings have a tendency to spread among the whole of the population, is true in London. The mortality in different districts varied from fifteen to twenty-nine in one thousand. The author points out the dangers in the outlying districts of London, which are imperfectly supplied with water and proper sewers. Over a million of population are without the proper sewerage area, and from this source London has more cause for fear than from any other.

This is true of many American cities, and is the fertile source of many epidemics and fevers. The author also urges that the municipal rule should include all the suburbs, supplying them with water, sewers, and sanitary supervision, then the mortality would diminish to twenty or less in one thousand. The mortality in London averaged twenty-one during the year. The maxima of mortality were in the coldest and hottest weather—January and August. This law prevailed to a large extent in all the towns of the kingdom. Diarrhœa was the most fatal prevalent disease in London; whooping-cough, small-pox, measles, and fevers, were the next most prevalent and fatal diseases. The deaths from violence have increased largely during the year. This is probably due to the carelessness of street drivers, and reckless disregard of crowds. Deaths from burning have declined unusually. The American reader will be surprised to learn that a very small proportion of violent deaths are from "gun shots." In our larger cities this is the most prevalent cause of violent deaths. The tables of death from fever show that fever matter (Typh) has an independent life of its own, and undergoes periodical developments. A history of thirty years back indicates successive years when fevers have been extremely prevalent and fatal. In 1864 it was epidemic, but in 1872 it had fallen to a low ebb. Three kinds of fever are prevalent over England—typhus, typhoid, and relapsing fever. Typhus has declined rapidly since 1869; typhoid has also declined, only more slowly, and relapsing fever remains about as before. In public institutions one person in every six dies, and this is increasing. In the hospitals more are dying, and fewer in workhouses and prisons. This is significant, and is explained by a late American writer to arise from the imperfect ventilation and the poisonous surroundings of these old hospital buildings, many of them over half a century old. The same writer claims that no hospital building that has been occupied over ten years is a proper place for sick persons. The walls, notwithstanding all fumigation, will retain poisonous fever germs and other dangerous animal matter. Mortality statistics of London, from 1840, indicate about 24 per cent. per one thousand. Divided up into five districts, in some an increase has been noticed, in others it has diminished.

Altogether the mortality has decreased at the rate of from one to one and a half per thousand; considering the steady increase of the population, this is very gratifying. The author says "disappointment may be felt that the mortality has not gone down to twenty per thousand permanently, but the reason is obvious, viz.: the water supply comes from the upper Thames, which drains a populous basin into which much impurity flows, and much of the sewerage is imperfect; these contribute to keep up the mortality. The low death-rate of last year is a favorable omen, yet the sanitary work of London is far from being complete. The mortality in the larger towns and cities of the kingdom is, in the aggregate, much higher. "In three cities where the population is very dense a high rate of mortality followed." "Density raises the mortality through the condensation of impurity; the quantity of air respired being equal in two populations, the quantity of impurity taken in bears a certain proportion to the strength of the noxious mixture. Density of population does not imply necessarily, but it does indicate density of zymotic impurity in most towns, and a resulting high rate of mortality." Increased precautions should follow in proportion to the density, to obviate its evils. The deaths for the year in these towns were over one thousand a week more than the healthy standard. Some of these towns show frightful mortality lists, and all are higher than that of London. One pleasing fact remains to be noted: nearly all the large capital cities of Europe, except in Russia, have a system of observations, recording deaths and fatal disease, by which we are able to make comparisons and trace causes, which the art and science of man may obviate. The great success which sanitary science has achieved in England gives promise of almost perfect immunity from such plagues as typhus, typhoid, diarrhœal affections, diphtheria, etc. If an improved system of ventilation, sewerage and good water diminishes the death-rate in Europe, and its old capital cities, what may we not expect from our young towns and cities, which are yet comparatively free from surface saturation with excreta of man and animals? This subject has a personal interest to all our surroundings, and particularly as we may now remedy these defects in a comparatively easy manner, when in later years the obstacles would be serious and almost insurmountable.—*Philadelphia Reporter.*

